# A multicenter effectiveness trial of QEEG-*informed* neurofeedback in ADHD: Replication and treatment prediction

**DOI:** 10.1016/j.nicl.2020.102399

**Published:** 2020-08-25

**Authors:** Noralie Krepel, Tommy Egtberts, Alexander T. Sack, Hartmut Heinrich, Mark Ryan, Martijn Arns

**Affiliations:** a Dept. of Cognitive Neuroscience, Faculty of Psychology and Neuroscience, Maastricht University, Maastricht, The Netherlands; b Research Institute Brainclinics, Brainclinics Foundation, Nijmegen, The Netherlands; c Dept. of Experimental Psychology, Utrecht University, Utrecht, The Netherlands; d neuroCare Group Netherlands, Nijmegen, The Netherlands; e neuroCare Group, Munich, Germany; f kbo-Heckscher-Klinikum, Munich, Germany; g neuroCare Group, Sydney, Australia

## Abstract

**Introduction:**

Quantitative Electroencephalogram*-*(QEEG-)informed neurofeedback is a method in which standard neurofeedback protocols are assigned, based on individual EEG characteristics in order to enhance effectiveness. Thus far clinical effectiveness data have only been published in a small sample of 21 ADHD patients. Therefore, this manuscript aims to replicate this effectiveness in a new sample of 114 patients treated with QEEG-informed neurofeedback, from a large multicentric dataset and to investigate potential predictors of neurofeedback response.

**Methods:**

A sample of 114 patients were included as a replication sample. Patients were treated with standard neurofeedback protocols (Sensori-Motor-Rhythm (SMR), Theta-Beta (TBR), or Slow Cortical Potential (SCP) neurofeedback), in combination with coaching and sleep hygiene advice. The ADHD Rating Scale (ADHD-RS) and Pittsburgh Sleep Quality Index (PSQI) were assessed at baseline, every 10th session, and at outtake. Holland Sleep Disorder Questionnaire (HSDQ) was assessed at baseline and outtake. Response was defined as ≥25% reduction (R25), ≥50% reduction (R50), and remission. Predictive analyses were focused on predicting remission status.

**Results:**

In the current sample, response rates were 85% (R25), 70% (R50), and remission was 55% and clinical effectiveness was not significantly different from the original 2012 sample. Non-remitters exhibited significantly higher baseline hyperactivity ratings. Women who remitted had significantly shorter P300 latencies and boys who remitted had significantly lower iAPF’s.

**Discussion:**

In the current sample, clinical effectiveness was replicated, suggesting it is possible to assign patients to a protocol based on their individual baseline QEEG to enhance signal-to-noise ratio. Furthermore, remitters had lower baseline hyperactivity scores. Likewise, female remitters had shorter P300 latencies, whereas boys who remitted have a lower iAPF. Our data suggests initial specificity in treatment allocation, yet further studies are needed to replicate the predictors of neurofeedback remission.

## Highlights

• QEEG-informed neurofeedback resulted in remission rates of 55%.
• In the total sample, non-remitters had higher hyperactivity scores at baseline.
• In women, non-remitters had longer P300 latencies.
• In boys, a low individual alpha peak frequency (iAPF) was associated with remission.

## 1 Introduction

Neurofeedback is a promising non-pharmacological treatment that has been well investigated in the treatment of ADHD. Neurofeedback can be considered a multi-factorial treatment including components such as reinforcement, coaching and direct feedback on brain-activity, in particular electrical brain activity (electroencephalogram; EEG). Not all EEG frequencies being trained have been shown to be efficacious. For example, training of the posterior alpha rhythm (8–13 Hz) has failed to show clinical benefit in either hyperkinetic syndrome (Nall, 1973) and epilepsy (Rockstroh et al., 1993), suggesting some *specificity* in the EEG parameter trained for clinically effective neurofeedback. Therefore, three well-investigated protocols (Sensori-Motor-Rhythm; SMR, Theta-Beta; TBR and Slow Cortical Potential; SCP) have been proposed as ‘standard neurofeedback protocols’ (Arns et al., 2014b). For these protocols meta-analyses have found support for clinical efficacy rated by parents (Cortese et al., 2016, Van Doren et al., 2019) as well as teachers, (Cortese et al., 2016). Foremost, clinical benefit of neurofeedback is maintained – with a tendency for further improvement over time – over 6–12 months follow-up periods, approaching clinical benefit obtained with psychostimulant medication (Van Doren et al., 2019). Holtmann and colleagues (2014) reported that SCP neurofeedback significantly decreased ADHD symptoms, however, when analyses were confined to probably blinded ratings, these effects were reduced to trend-level significance (Holtmann et al., 2014). A meta-analysis by Cortese et al. (2016) including also non-standard neurofeedback protocols reports that, when results are confined to probably blinded raters only, a previously significant result (as primarily reported by parents) becomes non-significant (although probably blinded ratings have some limitations, too (Van Doren et al., 2019)). Other studies also report contrasting support for the effectiveness of neurofeedback e.g. no difference between placebo and neurofeedback treatment, suggesting mechanisms of non-specificity (Logemann et al., 2010). Also, the benefits of neurofeedback for adults are still unclear, with mixed results (Mayer et al., 2015, Schönenberg et al., 2017). Therefore, the current study also aims to help build upon the body of knowledge currently investigating the effectiveness of neurofeedback. Next steps are: 1) to investigate the clinical effectiveness (also termed ‘Clinical Utility’), or the applicability, feasibility, and usefulness of the intervention in practice (American Psychological Association, 2002) and 2) to enhance clinical efficacy of this neurofeedback technique and to identify moderators, mediators, and predictors of remission, which is the primary focus of this manuscript.

In a small proof-of-concept study in 2012 by Arns and colleagues the clinical effectiveness of Quantitative Electroencephalogram (QEEG) *informed* neurofeedback was reported (Arns et al., 2012). In its essence, *QEEG-informed* neurofeedback is based on patient assignment to one of the above three ‘standard protocols’, taking the signal-to-noise ratio from their individual EEG into account. For example, it has been reported that patients with high theta (low beta), and high theta/beta ratio (TBR) respond better to theta/beta neurofeedback (Gevensleben et al., 2009). However, high theta and a high TBR are not present in all children with ADHD, but are consistently found in 1/3rd of children with ADHD (Arns et al., 2013, Bussalb et al., 2019). Therefore, cases with high theta will be preferentially assigned to TBR-neurofeedback. In addition, the exact theta frequency band that will be trained is individualized (i.e. 4–6 Hz, or 5–8 Hz) to increase signal-to-noise ratio and thus the specificity of the feedback. In cases with no clear excess of theta, patients will be treated with SMR or SCP neurofeedback, depending on trainability in that respective frequency band (i.e. in cases of excess 11–13 Hz Mu rhythm activity in sensori-motor regions overlapping with the 12–15 Hz SMR band, SCP neurofeedback is preferred over SMR neurofeedback). In this way, virtually every patient will be treated with one of the ‘standard neurofeedback’ protocols. In addition, a second protocol can be added based on the presence of other EEG hypovigilance markers such as excess frontal alpha (Arns and Kenemans, 2014, Sander et al., 2010) or spindling excessive beta, often associated with impulse control problems (Arns et al., 2015). Arns et al. (2012) demonstrated that QEEG-informed neurofeedback was effective in decreasing ADHD symptoms and, importantly, response rates and effect sizes surpassed those of meta-analyses where one protocol was applied to the whole population (response rate of 76% (≥50% symptom reduction)) and large effect sizes for inattention and hyperactivity were observed (Arns et al., 2012). However, these results still require replication.

In an effort to optimize treatment, predictors, moderators, and mediators of treatment success are often considered. Although not widely studied, some researchers have attempted to identify these. For example, EEG profiles have been proposed as a potential moderator of neurofeedback response in terms of clinical improvement. Specifically, EEG-subtypes may be independent of diagnostic status (Clarke et al., 2011, Clarke et al., 2001, Clarke et al., 2003) and preselecting individuals for a particular type of neurofeedback based on their EEG profile may result in greater clinical improvements (Gevensleben et al., 2009). Other results from the NIMH-MTA trial, including three arms of treatment – behavioral, medication, and a combination thereof – found as moderators that youth with ADHD and comorbid anxiety disorder had a better response to behavioral or combined therapy (Hinshaw et al., 2015). Similarly, the MTA trial also found that those with anxiety and comorbid conduct or oppositional defiant disorder responded better to combined therapy (Hinshaw et al., 2015). For adults, one medication study found that individuals that were younger, female, and had higher baseline scores had greater clinical improvements (Weiss et al., 2010). As mediators, Hinshaw and colleagues identified that, in combined therapy only, improved parenting skills over the course of treatment was linked to decreased aggressive and disruptive behavior in their children as well as increased social skills (Hinshaw et al., 2015). Interestingly, another study also highlighted the importance of parenting style for successful (combined EEG biofeedback and) methylphenidate treatment (Monastra et al., 2002).

Therefore, the purpose of this study is twofold. Firstly, the aim is to replicate the clinical effectiveness of QEEG-*informed* neurofeedback in clinical practice, as reported by Arns and colleagues in 2012. It was hypothesized that the effectiveness would not deviate significantly in the new sample relative to the 2012 results. Second, baseline clinical as well as neurophysiological variables (EEG and ERP) were examined as moderators, mediators (Baron and Kenny, 1986, Kraemer et al., 2002) and predictors of neurofeedback (non-)remission. A recent study by Arns and colleagues found that, in boys only, a lower iAPF was indicative of MPH nonresponse (Arns et al., 2018), so in the current study iAPF will also be examined. Analyses will be primarily focused on remission, rather than response. This was done because remission is considered a more clinically relevant endpoint as it implies a loss of diagnostic status (Steele et al., 2006), instead of merely a decrease of symptom presentation, and thus provides a clearer distinction between groups. However, to elucidate the effect of remission versus response, sensitivity analyses were also performed using response as a clinical endpoint, to further crystallize the (potential) differences between the response and remission and potential predictors thereof.

## 2 Methods and materials

### 2.1 Participants

The full sample consisted of 136 patients for the first analysis, 115 of which were acquired in the new sample and 21 that were already reported in Arns et al. (2012). This study was an open-label, naturalistic, multi-site study. Given the open-labelled nature of this study, treatment was performed as usual and the analyses were performed post-hoc. Therefore, this study was not reviewed by an independent ethics committee. Patient data were collected from five clinics, two in the Netherlands (neuroCare Group Nijmegen & neuroCare Group The Hague), one in Germany (neuroCare Group Munich) and two clinics in Australia (neuroCare Group Frenchs Forest and neuroCare Group Sydney). Data were collected between August 2008 and May 2018. Patients were screened for inclusion and included in case of an ADHD or ADD diagnosis (as confirmed by the MINI Diagnostic Interview or by a qualified clinician), or when ADHD-RS scores on either scale (ATT or HI) was equal to or higher than 6 (for adults a cut-off of 5 or higher was used, in line with current DSM-5 diagnostic requirements). The ADHD Rating Scale (ADHD-RS, (Kooij et al., 2008)) and the Pittsburgh Sleep Quality Index (PSQI, (Buysse et al., 1989)) were obtained at intake, every 10th session, and at outtake. If applicable, the Beck Depression Inventory (BDI-II-NL) and Depression, Anxiety, and Stress Scale (DASS) were assessed at intake, every 10th session, and at outtake as well. All patients signed an informed consent before treatment was initiated. In the case of children younger than 18, caregivers signed the informed consent form. Patients arrived at the clinic referral-based and received (partial) financial support from the government or health insurance, although the majority of treatments was self-paid.

### 2.2 QEEG

QEEG recordings were performed in accordance with the standardized methodology as developed by Brain Resource Ltd. (details of which can be found here (Arns et al., 2016)), of which reliability, validity, and across site-consistency has been published elsewhere (Clark et al., 2006, Paul et al., 2007, Williams et al., 2005). In short, a 26-channel recording based on the 10–20 electrode international system using the Quickcap was administered in a standardized room. Data were referenced to averaged mastoids with a ground at AFz. Horizontal and vertical eye movements were controlled for. Skin resistance was <10 kΩ for all electrodes. Data were offline corrected for EOG. The sampling rate was 500 Hz for all electrodes. A low pass filter above 100 Hz was applied prior to digitization. The EEG test battery consisted of nine tasks in total, three of which are considered in the current study: a 2-minute Eyes Open (EO) task, a 2-minute Eyes Closed (EC) task, and a 6-minute auditory oddball (ODDB) task.

ERP scoring is thoroughly described by van Dinteren and colleagues (2014). ERP’s were deduced from the ODDB task, in which a series of high- and low-pitched tones were quasi-randomly presented (the only constraint being that two high-pitched tones cannot occur right after each other), and the patient was asked to press a left- and right-handed button simultaneously at the high-pitched tones. ISI was 1 s. For ERP extraction, windows around the target stimuli of −300 ms to 700 ms were examined. Data were 25 Hz low-pass filtered and baselined to the relative 300 ms pre-stimulus window. Peak components were determined according to maximal response within specific latency intervals. This gave amplitudes and latencies for points N200 and P300 (Arns et al., 2008, Bahramali et al., 1999, Lim et al., 1999, Williams et al., 2005). In this study, the primary focus will be on P300.

iAPF determination was based on prior studies (Arns, 2012, Arns et al., 2018) and consisted of the following steps: 1) Fast Fourier Transform to both EO and EC conditions using 2000 ms segment epochs, 2) the difference between EO and EC power spectra was calculated (by subtracting EO from EC) in order to distinguish the alpha power (6–13 Hz) by its known suppression from EC to EO, and 3) the iAPF was determined by identifying the maximum value between 6 and 13 Hz.

### 2.3 Neurofeedback treatment

Treatment of patients was identical to treatment as reported in 2012 and 2014 by Arns and colleagues (Arns et al., 2012, Arns et al., 2014a). In short, before treatment was started patients were assessed using the QEEG, through which the choice for a QEEG-informed neurofeedback treatment protocol was derived. In some cases, neurofeedback protocol was adjusted according to the patient’s needs. SMR neurofeedback was performed using a 12–15 Hz reward at central locations (C3, Cz, or C4). The TBR protocol consisted of a reward in the beta frequency range (e.g. 20–25 Hz) at midline sites Fz, FCz, or Cz, in addition to inhibition of theta power. The only difference with the procedure reported in 2012 and 2014, was that in the current sample neurofeedback treatment was complemented with sleep hygiene management and coaching.

The choice for a particular neurofeedback protocol was based on the QEEG assessed during EO and EC:

• Theta/(beta) protocol: when excess fronto-central slowing was observed. Only beta reward if beta was not elevated or beta spindles were not present. Only midline sites (Fz, FCz, Cz).
• SMR/SCP protocol: no clear QEEG deviations and/or sleep problems.
• Low-voltage EEG: SMR/SCP neurofeedback and/or alpha-uptraining during EC at Pz.
• Frontal Alpha protocol: when excess fronto-central alpha (mostly EO) was observed. Beta reward as per Theta/(beta) protocol. Only midline sites (Fz, FCz, Cz); mostly in adult ADHD.
• Beta-downtraining protocol: when beta spindles or excess beta was present, the specific frequency of this excess beta (spindles) was downtrained on the frontocentral site with maximal beta-spindle power.

All protocols employed EMG inhibits, where EMG (55–100 Hz) had to be kept below 5–10 μV.

Sessions were performed by a master’s level psychologist specialized in neurofeedback, trained and accredited by the last author, and took place 2–3 times a week. 20–30-minute sessions were administered, offered in blocks of five minutes each, with a minimum one-minute break in between blocks. Threshold parameters were set to achieve 25–40% effective reinforcement. For SMR treatment, the time-above-threshold was set at 0.2–0.5 s. Equipment used to provide visual and auditory feedback consisted of Brainquiry PET 4.0 (Brainquiry B.V., Nijmegen, the Netherlands) and BioExplorer software (CyberEvolution, Inc., Seattle, USA) for frequency neurofeedback. SCP Neurofeedback was provided using a Theraprax system (neuroConn, Ilmenau, Germany).

### 2.4 Data analysis

ADHD patients were categorized into four groups, according to outtake data, or the last available assessment (Last Observation Carried Forward, LOCF) (based on (Arns, 2012)):

- *Response (R)*: either 25% (R25 (Steele et al., 2006)) or 50% (R50) or more reduction in ADHD-RS Inattention scale (ATT) or Hyperactivity/Impulsivity scale (HYP). Both criteria were used to ensure comparability with other studies (e.g. (Strehl et al., 2017).
- *Remission:* remission (i.e. loss of diagnostic status) was defined as an ADHD-RS item mean of ≤1.00 (Steele et al., 2006, Swanson et al., 2001).
- *Drop-out (DO)*: when a patient did not take more than 20 sessions and could not be classified as a responder. In this case, the patient was not included in the analyses.
- *Non-responder (NR)*: a patient who had more than 20 sessions and did not meet the criteria for being a responder.

## 3 Statistics

To estimate the efficacy of QEEG-informed neurofeedback as a treatment for ADHD symptomatology, the response rates of the 2019 sample were compared to those of the 2012 sample, using Chi-square statistics. To study possible differences between the 2012 and 2019 sample as well as differences in response for children vs. adults, males vs. females, and protocol specific effects, a repeated measures ANOVA with Time (pre-, halfway-, and postintervention measurements) as a within-subject factor and Sample (2012 and 2019), Sex (female and male), Protocol (SMR, TBR, and other (specifically: SCP and protocols other than SMR/TBR)), and Age group (children and adults) as between-subject factors was performed. Only main effects of Time, Sample, Sex, Age Group and Protocol and interactions with Time were considered. Lastly, baseline clinical and neurophysiological variables were examined for their value in predicting neurofeedback (non-)remission. In the current study, predictors are defined as variables that are associated with better or worse treatment outcome (followed from Hinshaw et al. (2015), in accordance with Kraemer et al. (2002)). For clinical variables, a GLM Univariate using a potential predictor as a dependent variable, age as a covariate, and Protocol (SMR, TBR, and other), Sex (female and male), and Remission (remission and no remission) were used as between-subject factors, was performed. For neurophysiological variables, the different components of ODDB ERP’s were examined. This was done using a repeated measures ANOVA with Site (Fz, Cz, Pz) as a within-subject factor, and Protocol (SMR, TBR, and other), Sex (female and male), and Remission (remission and no remission) as between-subject factor, while covarying for age. A similar approach was taken for iAPF, in which case iAPF was examined using a repeated measures ANOVA using Site (Fz, FCz, Pz, Oz) as a within-subject factor and Protocol (SMR, TBR, and other), Sex (female and male), and Remission (remission and non-remission) as between-subject factors, while covarying for age. For TBR the same analyses as for iAPF were performed, however, in the within-subject factor Site the sites Fz and Cz were used instead of Fz, FCz, Pz, and Oz. TBR analyses were also repeated for SMR and TBR protocols separately, given the probable selection bias because of QEEG-informed neurofeedback. Predictors were examined for their predictive utility by performing a discriminant analysis and investigating the Receiver Operator Curve (ROC). A side-track of this study entails a possible association between hyperactivity and sleep breathing problems, based on (Vollebregt et al., 2019, June 12). Vollebregt and colleagues found that children with sleep breathing problems exhibited increased levels of hyperactivity. In the current study, this association will be tested by performing a bivariate Spearman correlation between SBD and HYP. Potential mediator/moderator analyses were performed based on non-null findings. Mediator and moderator analyses were performed in accordance with Baron and Kenny (1986) and (Kraemer et al., 2002). For mediation to occur, the following criteria should be met: 1) the independent variable should significantly affect the presumed mediator, 2) the presumed mediator should significantly affect the dependent variable, and 3) when paths described in 1) and 2) are controlled for, the previously significant association between the independent and dependent variable should no longer exist (Baron and Kenny, 1986). Kraemer et al. (2002) added additional requirements for mediation in a clinical setting, being that 1) a mediator should measure a change or event during treatment, 2) the mediator must correlate with treatment choice, and 3) should have a main or interactive effect on the outcome. If mediation analyses were to be performed, partial correlations were run, while controlling for (one of) the potential mediator(s). On the other hand, Kraemer et al. (2002) describe a moderator of treatment efficacy such that a moderator 1) must be gathered at baseline or prior to randomization and 2) explains individual differences in treatment efficacy, meaning that the effect of treatment depends on the value of the moderator (Kraemer et al., 2002). In case of moderator analyses, the individual potential moderators and the interaction between the two (moderator_A_ * moderator_B_) were used in a linear regression as independent variables, while the variable of interest was used a dependent variable. In case of mediator analyses, partial correlations were run, correlating two out of three variables of interest, while controlling for the remaining variable. All predictive analyses, only relevant Remission effects and interactions were considered. Effect sizes reported are Cohen’s *d* and were calculated using the following formula: $d=\frac{m1-m2}{\sqrt{\frac{\left(s1^{2}+s2^{2}\right)}{2}}}$. Error bars represent ±2SE. All analyses were performed in IBM SPSS Statistics 25 for Macintosh.

## 4 Results

The full sample consisted of 136 patients for the first analysis, 114 (excluding 1 DO and the 21 already reported in (Arns et al., 2012)) were included to replicate the initial response to treatment and outcomes were statistically compared to the results of Arns and colleagues in 2012). For further analyses the full sample was used. The demographics of the total sample, the 2019 and the 2012 sample can be found in Table 1. Note: given the clinical focus of the paper, medication usage was not controlled for.

**Table 1:** Descriptive statistics for the total sample with means and (SD), and separately for 2019 and 2012 sample. No significant differences were found (p ≥ 0.055).

	**Total sample**	**2019 sample**	**2012 sample**
**Age**	24.9 (14.9)	24.0 (14.6)	30.0 (16.2)
**Number of sessions**	32.3 (10.1)	32.0 (8.6)	33.6 (16.1)
**Protocol (n, (%))**			
*SMR*	84 (61.8)	69 (60.0)	15 (71.4)
*TBR*	27 (19.9)	25 (21.7)	2 (9.5)
*Other*	25 (18.4)	21 (18.3)	4 (19.0)
SCP	9	9	0
**Males (n, (%))**	89 (65.4)	76 (66.1)	13 (61.9)
**Adults (n, (%))**	80 (58.8)	66 (57.4)	14 (66.7)
**ADHD total**	12.4 (3.1)	12.5 (2.9)	11.5 (4.1)
ADHD total post	4.6 (4.6)	4.8 (4.7)	3.6 (3.6)
**ADHD Hyperactivity (HYP)**	5.5 (2.4)	5.6 (2.2)	4.7 (2.9)
ADHD Hyperactivity post	2.0 (2.3)	2.1 (2.3)	1.3 (2.3)
**ADHD Inattention (ATT)**	6.9 (1.8)	6.9 (1.7)	6.8 (2.0)
ADHD Inattention post	2.6 (2.7)	2.6 (2.8)	2.3 (2.2)
**PSQI**	7.7 (4.2)	7.4 (4.1)	9.6 (4.6)
PSQI post	4.6 (3.1)	4.5 (3.0)	5.6 (3.4)

### 4.1 Clinical outcome

Remission and response rates of the current sample (average age: 24.0; range 6–68 yrs; 76 males) were 54.8% remission, and 70.4% and 85.2% response for R50 and R25 criteria respectively. This was not significantly different (R50: *χ*^2^(1) = 1.428, p = 0.232) relative to the 2012 sample. Given clinical response was the same in both samples, the pooled remission and response rates in the full sample of 136 patients were 57.4% remission and 71.3% and 83.8% response for R50 and R25 criteria respectively.

### 4.2 Moderating effects

**Fig. 1:**
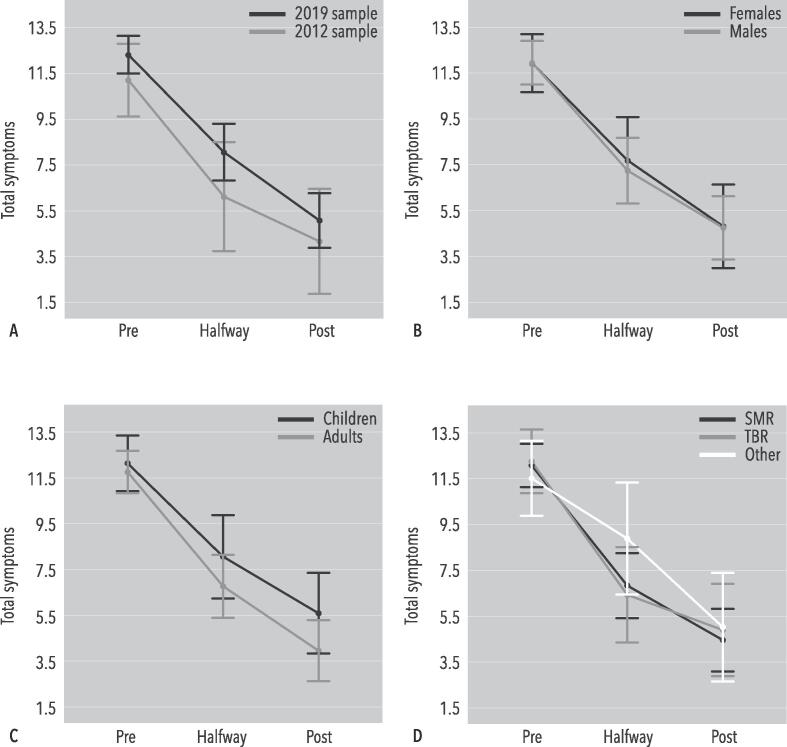
A repeated measures ANOVA using Sample (2012 v. 2019), Sex (female v. male), Age group (children v. adults), and Protocol (SMR, TBR, other) as between-subject factors. Total ADHD-RS symptoms were used as a within-subject factor (pre-, halfway-, and post-measurements). The error bars represent 2SE. Analyses showed a significant effect of Time (F(2,114) = 48.171, p < 0.001; *d* = 1.97), but no other significant interactions or main effects were observed.

A repeated measures ANOVA showed a significant effect of Time (F(2,114) = 48.171, p < 0.001; *d* = 1.97). No other significant interactions or main effects were observed, thus clinical response was not moderated by age-group, sex and neurofeedback protocol and no differences between the 2012 and current sample were found. These effects are visualized in Fig. 1.

### 4.3 Predictors of neurofeedback (non-)remission

**Fig. 2:**
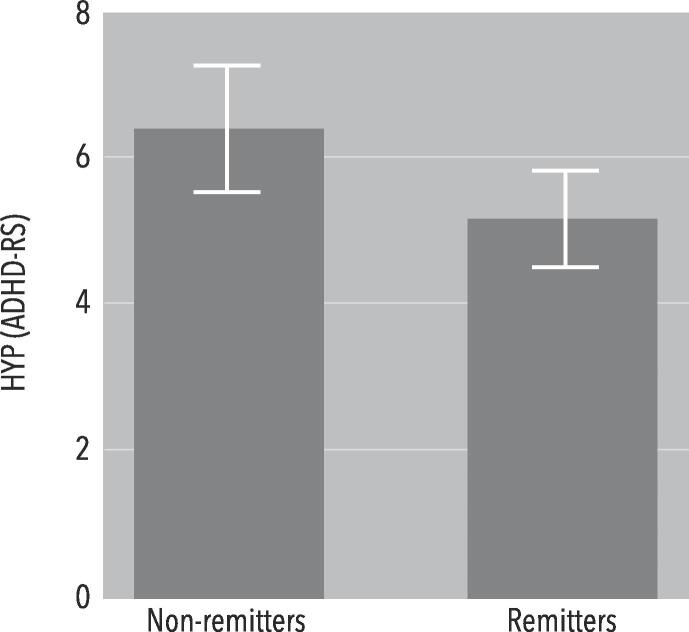
Bar graph of HYP scores, separated for remitters and non-remitters. A GLM Univariate analysis showed a significant main effect of Remission (F(1,114) = 5.095, p = 0.026; *d* = 0.56).

GLM Univariate analyses showed no significant main or interaction effects for ADHD total symptoms, nor for ATT, PSQI total score, HSDQ total score, insomnia, parasomnia, CRSD, hypersomnia, RLS-PLMD, or SBD (p≥0.102). However, for HYP there was a significant main effect of Remission (F(1,114) = 5.095, p = 0.026; *d* = 0.56). Thus, remitters had lower HYP scores at baseline (Fig. 2). Using HYP in a discriminant analysis yielded a significant model (p = 0.004; Wilks’ Lambda = 0.934; Chi-square = 8.466; df = 1; AUC = 0.635).

**Fig. 3:**
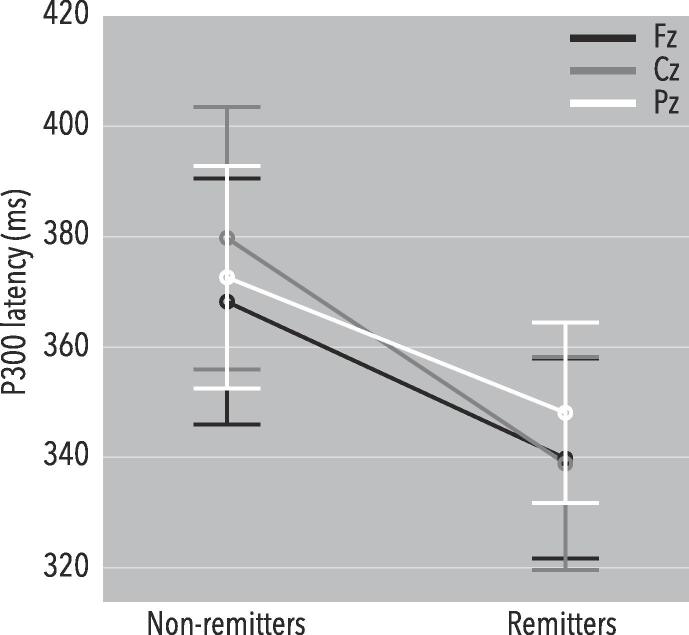
P300 latencies separated by remission. A repeated measures ANOVA showed that female remitters had a significantly shorter P300 latency (F(1,25) = 5.570, p = 0.026; *d*_Fz_ = 0.87, *d*_Cz_ = 0.85, *d*_Pz_ = 0.51).

For ERP variables, a repeated measures ANOVA showed no significant main or interaction effects for N200 amplitude and latency, and P300 amplitude. For P300 latency, a significant Site X Sex X Remission effect was found (F(1.713,154.200) = 3.235, p = 0.050), and a main effect of Remission (F(1,90) = 5.082, p = 0.027). There also was a significant main effect of Remission X Sex (F(1,90) = 3.958, p = 0.050). Splitting by Sex, in women there was a significant main effect of Remission (F(1,25) = 5.570, p = 0.026; *d*_Fz_ = 0.87, *d*_Cz_ = 0.85, *d*_Pz_ = 0.51; Fig. 3), yet for men no such effect was observed. Using P300 latency at Fz in a discriminant analysis yielded a significant model (p = 0.025; Wilks’ Lambda = 0.844; Chi-square = 5.007; df = 1; AUC = 0.743). Thus, female remitters had shorter P300 latencies.

For TBR, no significant results were obtained. Thus, TBR was not related to remission.

**Fig. 4:**
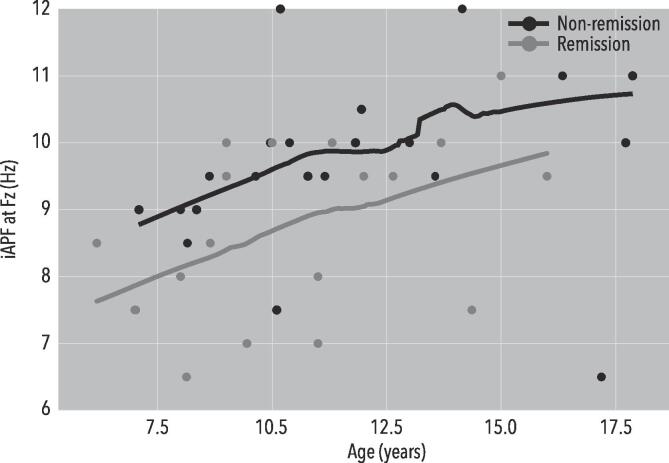
A Loess-fit for iAPF and Age, separated for Remission and Non-remission, for male youngster only. A repeated measures ANOVA showed a significant main effect of Remission (F(1,37) = 4.534, p = 0.040; *d*_Fz_ = 0.78, *d*_FCz_ = 0.68, *d*_Pz_ = 0.42, *d*_Oz_ = 0.66).

For iAPF analyses, a repeated measures ANOVA yielded no significant results. Based on earlier work (Arns et al., 2018) and a directed hypothesis, the analysis was repeated in a selected sample of boys (average age: 11.1; age range: 6–18) only. The resulting sample consisted of 45 boys, three of which were excluded based on missing data (21 remitters, 21 non-remitters). A One-Way ANOVA showed no significant Age difference between remitters and non-remitters (F(1,40) = 1.244, p = 0.271). A repeated measures ANOVA using only Remission as a between-subject factor yielded a significant main effect of Remission (F(1,37) = 4.534, p = 0.040; *d*_Fz_ = 0.78, *d*_FCz_*=*0.68, *d*_Pz_*=*0.42, *d*_Oz_*=*0.66). Using iAPF at Fz in a discriminant analysis yielded a significant model (p = 0.019; Wilks’ Lambda = 0.863; Chi-square = 5.508; df = 1; AUC = 0.694). The iAPF for remitters and non-remitters for Fz was 8.7 Hz vs. 9.7 Hz, respectively. This can be observed in Fig. 4. This indicates that, in the group of boys only, remitters had a lower mean iAPF.

Concluding, apart from HYP at baseline, no other clinical variables served as predictor for neurofeedback (non-)remission. From the ERP analyses P300 latency for women emerged as a predictor, however, no other components of P300 showed to be useful. TBR and iAPF both showed to be not useful in predicting neurofeedback remission in the full sample. However, a subsample analysis showed a significant result for boys, where a slow iAPF was associated with remission.

### 4.4 Post-hoc exploratory analysis

Based on the above results and earlier work indicating an association between HYP and SBD (Vollebregt et al., 2019, June 12), specific relations between variables were explored to further elucidate the direction of effects for HYP as a predictor.

In (Vollebregt et al., 2019, June 12) a relation between hyperactivity and SBD in children was found, thus suggesting hyperactivity symptoms can be caused by SBD, and thus the association between SBD and reduced clinical response, could be mediated by the presence of HYP. To test this further in the current sample (children only), a bivariate Spearman correlation between HYP and HSDQ SBD was performed. A significant correlation was found (r(35) = 0.353, p = 0.038; r^2^ = 12.4%). Similarly, a bivariate Pearson correlation between HYP and clinical response showed to be significant (r(50) = 0.314, p = 0.026; r^2^ = 9.9%). However, a bivariate Pearson correlation between SBD (LOG-transformed) and clinical response was non-significant (r(32) = 0.036, p = 0.844; r^2^ = 0.1%). Because of the directionality and assumed working mechanism between SBD, HYP, and remission, a mediation analysis was performed. A partial correlation between HYP and clinical response, while controlling for SBD (LOG-transformed), yielded a significant correlation (r(29) = 0.377, p = 0.037; r^2^ = 14.2%). A partial correlation using SBD (LOG-transformed) and clinical response also showed a non-significant association (r(29) = −0.076, p = 0.684; r^2^ = 0.6%), leaving both associations unchanged. It was also tested whether children with or without SBD complaints had different outcomes on ATT or HYP %change. A Mann-Whitney U using SBD (with or without complaints) as a between-subject factor and ATT and HYP %change as dependent variables was performed. This yielded no significant results for ATT (Mdn_remitters_ = 100.0, Mdn_non-remitters_ = 25.0, U = 127.5, Z = −0.754, p = 0.451), nor for HYP (Mdn_remitters_ = 100.0, Mdn_non-remitters_ = 37.5, U = 120.5, Z = −0.995, p = 0.320). Thus, even though that SBD seems to be related to hyperactivity and hyperactivity seems to be related to remission, there seems to be no interaction between HYP and SBD.

### 4.5 Sensitivity analyses

Given the overrepresentation of the SMR protocol in the current sample and the primary focus on remission, further analyses were performed to investigate the specificity of the obtained results. That is, focusing only on the significant results obtained in the main manuscript*,* analyses were repeated using Response (50%) as a between-subject factor instead of Remission. Analyses were also performed in the SMR group alone. The performed analyses are identical to the above. In the SMR-specific analyses, Remission was used as a between-subject factor. Details of the analyses can be found in the supplement.

Summarizing the results in the supplement, HYP and P300 did not emerge as predictors of non-response. For iAPF, the difference was not significant, albeit the direction of the result was the same and the effect size was similar to the one observed in the main manuscript. The SMR analyses showed no significant effects, but the directions of the effects and effect sizes were similar to the ones observed in the main manuscript.

## 5 Discussion

This paper aimed to replicate the clinical effectiveness of QEEG-informed neurofeedback, as reported in (Arns et al., 2012). Also, potential moderators, mediators, and baseline behavioral and neurophysiological variables as predictors were examined of neurofeedback remission.

Clinical effectiveness of QEEG-*informed* neurofeedback was replicated, meaning that the current response and remission rates were not significantly different from those reported in 2012. Furthermore, hyperactivity emerged as a potential predictor of neurofeedback non-remission, specifically, non-remitters had higher baseline hyperactivity scores. Additionally, females who had a faster P300 latency were more likely to be remitters, whereas boys who remitted had lower iAPF as compared to those who did not. Lastly, SBD seemed to be significantly related to hyperactivity, however, hyperactivity does not seem to mediate the association between remission and SBD.

The effectiveness of this study yielded equal or larger effect sizes as reported by a meta-analysis that focused on neurofeedback randomized controlled trials (Cortese et al., 2016), and demonstrates similar remission rates and effect sizes compared to the NIMH-MTA Medication Management treatment (The MTA Cooperative Group, 1999). While the design of the current study was an open-label trial, it provides important information regarding effectiveness or ‘Clinical Utility’ meaning the applicability, feasibility, and usefulness of the intervention in clinical practice. This construct is designed to assess the generalizability of the intervention into everyday clinical practice (American Psychological Association, 2002). For example, when considering clinical efficacy for methylphenidate in the treatment of ADHD as established in the MTA trial, remission rates of 56–68% were reported for the medication arms, while the results of the large international multicenter iSPOT-A effectiveness study yielded a 31% remission rate and a 33% smaller effect size for effectiveness obtained in clinical practice. Furthermore, in a study where the MTA medication algorithm was followed, a 44% smaller effect size was reported (Geladé et al., 2018), illustrating that clinical utility is equally important in the consideration of generalizability of clinical effects into clinical practice. Therefore, this study demonstrates that, across the five clinics involved, the effectiveness of neurofeedback translates well into practice. Potential reasons as to why the current study found greater effect sizes include the assumed specificity of QEEG-*informed* neurofeedback and the targeted frequency band and the increased emphasis on sleep hygiene management, however, the exact reasons should be investigated in further controlled studies.

This study suggests that non-remitters were characterized by higher hyperactivity scores at baseline, albeit this finding was not found in the sensitivity analysis for response (R50). This is most likely due to the definition of remission, requiring full symptom resolution in absolute terms (item mean ≤ 1.0) opposed to response, which is a relative metric, and thus less sensitive to initial severity. The current result is in line with the notion symptoms of hyperactivity may be less sensitive to the effects of neurofeedback (Arns et al., 2009, Holtmann et al., 2014). Our results further indicated that SBD was significantly related to hyperactivity, and hyperactivity was associated with non-remission, yet SBD was not related to remission. Hyperactivity did not seem to act as a mediator in this working mechanism. This is in line with (Chervin and Archbold, 2001), who found that children with or without SBD scored equally high on hyperactivity. However, a recent meta-analysis found that people presenting symptoms of SBD are at an increased risk of developing complaints of inattention and hyperactivity, and therefore it is argued that people showing ADHD complaints should be screened for SBD (albeit the age groups only concerned children and adolescents and the overall effect showed a medium effect size (Hedges’s g = 0.57) (Sedky et al., 2014).

For P300, prior studies have primarily focused on P300 amplitude, whereas P300 latency is less well studied. Although the majority of studies generally concern a small sample size, results seem to converge on a prolonged P300 latency in children with ADHD (Sanfins et al., 2017, Sunohara et al., 1997, Tsai et al., 2012, Yamamuro et al., 2016b), albeit support for this seems to be less clear in adults (Szuromi et al., 2011). Interestingly, a recent study by Chi and colleagues found that parents with ADHD offspring had longer P300 latencies (Chi et al., 2019). P300 latency deviances may also not solely occur in ADHD (e.g. (Degabriele and Lagopoulos, 2009, Gao and Raine, 2009, Qiu et al., 2014, Simons et al., 2011)). An important role in the presentation of P300 latency and amplitude is age, specifically, around the age of 16 P300 amplitude tends to decrease, whereas the latency tends to increase after the age of 22 (van Dinteren et al., 2014). Yet, since in P300 analyses age was used as a covariate, it is not expected that age might explain the current results. Regarding prognostics, normalization of ERP variables after pharmacological treatment has been reported (Ozdag, et al.; Yamamuro et al., 2016a), yet P300 has not yet been evaluated as a predictor per se. As to why the current effect was specifically observed in women is not entirely clear. Some sex differences have been reported (Bakos et al., 2016, Nanova et al., 2008), but a recent systematic review showed that the effect of sex on P300 latency is minimal to none (Melynyte et al., 2018). Also, sex specific concerns in the presentation of ADHD may be considered (Nussbaum, 2012). Importantly, given the above variance in available literature, this effect may be spurious and therefore requires thorough further investigation and replication.

Interestingly, in the current sample, boys who remitted to neurofeedback exhibited a lower frontal iAPF, whereas in (Arns et al., 2018) the opposite was found for treatment with methylphenidate. These results may indicate frontal iAPF as a stratification biomarker to stratify, or differentially assign boys between two effective treatments (in this case low iAPF implicates neurofeedback and high iAPF implicates MPH), given the opposite association. However, further studies will need to prospectively test and replicate this as a possibility to further optimize and individualize ADHD treatments.

Concluding, the clinical effectiveness of QEEG-informed neurofeedback was replicated, and clinical benefit was the same for males vs. females, children vs. adults and irrespective of the protocol used. Hyperactivity, iAPF, and P300 may serve as potential predictors of neurofeedback (non-)remission, although these findings still need to be replicated and tested for robustness.

### 5.1 Limitations

This study was based on a naturalistic, open-label design. While this can be viewed as a strength of the study (effectiveness, results translate into clinical practice), this is also a weakness of the study, since no control condition was used and effect sizes obtained are sometimes higher in such designs. This also means that potential non-specific mechanisms subjective to treatment as usual (e.g. structured environment, regular intervals of training) may have impacted clinical efficacy and thereby the current results. Future, randomized controlled studies should further investigate the added effect of assigning people to an individualized neurofeedback protocol, such as the QEEG-*informed* neurofeedback presented here. Furthermore, patients in this study received treatment as usual, that included additional coaching and managing of sleep hygiene based on the patient’s individual needs. Also, medication usage was not controlled for in the current analyses. Importantly, the majority of the current sample had already sought treatment for ADHD symptoms, yet had insufficient relief from their sypmtoms and therefore sought additional treatment options. Of the total (n = 136) sample, 43 patients did not use any medication at all. The remaining part used a combination of stimulant medication, sleep medication (among which melatonin), benzodiazepines, and antidepressant medication. To investigate potential medication effects, post-hoc analyses were repeated on the sample free of medication. The direction of the results remained unchangend, however, some of the results did not reach significance. Note that sample sizes were significantly reduced given the restriction of no medication, thereby complicating interpretation. Another limitation is that this study only considered baseline clinical and neurophysiological data. This means that changes in clinical assessment may have been the result of neurophysiological changes due to neurofeedback treatment (or vice versa). Indeed, Arns et al. (2012) found in their initial study that, after SMR treatment, P300 amplitude had increased and SMR power had decreased. Yet, this sample size was small and the current study does not have the necessary post EEG measurements to test this question. Future, well-powered studies entailing post EEG’s should focus on this issue. Another issue (although perhaps not a limitaiton per se) is that the Contingent Negative Variation (CNV) was not considered in this study. The CNV was not considered because the SCP neurofeedback sample was small (n = 9) and, given that several studies have found the effect in SCP neurofeedback (Gevensleben et al., 2014, Heinrich et al., 2004) (Mayer et al., 2016), unsuitable for data analysis. Similarly, the CNV is typically extracted at more than 1000 ms after cue onset. Given that the oddball paradigm used in this study had an ISI of 1000 ms, this paradigm was unsuitable for CNV extraction. However, some studies have shown that the CNV shows potential to be used in clinical practice, and therefore future studies may investigate this issue further. Lastly, even though the total sample is 136 and thus sufficiently statistically powered, zooming in on subgroups resulted in a substantial decrease in sample size, resulting in the smallest sample size of 11 (females, children).

## Declaration of Competing Interest

The authors declare that they have no known competing financial interests or personal relationships that could have appeared to influence the work reported in this paper.
