# Supplementary material for: A multicenter effectiveness trial of QEEG-informed neurofeedback in ADHD: Replication and treatment prediction
Source: Neuroimage Clin. 2020 Aug 25;28:102399. doi: 10.1016/j.nicl.2020.102399 (PMC7481574; doi:10.1016/j.nicl.2020.102399)
Supplement: Supplementary data 1 [file mmc1.pdf]

Sensitivity analyses

Given the overrepresentation of the SMR protocol in the current sample and the primary focus on remission, further analyses were performed to investigate the specificity of the obtained results. That is, analyses were repeated using Response (50%) as a between-subject factor instead of Remission, and the analyses were also performed in the SMR group alone.

Effect sizes reported are Cohen's *d* and were calculated using the following formula: $d = \frac{m1-m2}{\sqrt{\frac{(s1^2+s2^2)}{2}}}$. Error bars represent ±2SE. All analyses were performed in IBM SPSS Statistics 25 for Mac.

In a univariate analysis using Response (response and non-response) as a between-subject factor, age as a covariate, and HYP as a dependent variable, a non-significant main effect of Response was observed (Figure 1A; $F(1,130)=.405$, $p=.526$; $d=-.10$). This is in contrast with the obtained finding in the main manuscript. For P300 latency, focusing on women only, a repeated measures ANOVA using Response (response and non-response) as a between-subject factor, age as a covariate, and Site (Fz, Cz, Pz) as a within-subject factor, showed no significant main effect of Response (Figure 1B; $F(1,30)=.039$, $p=.845$; $d_{Fz}=.16$, $d_{Cz}=-.21$, $d_{Pz}=-.14$), potentially indicating a severity-specific effect of P300 latency. For iAPF analyses, confining to boys only, a repeated measures ANOVA using Response (response and non-response) as a between-subject factor and iAPF (Fz, FCz, Pz, Oz) as a within-subject factor yielded a non-significant effect of Response (Figure 1C; $F(1,40)=2.512$, $p=.121$; $d_{Fz}=.44$, $d_{FCz}=.29$, $d_{Pz}=.63$, $d_{Oz}=.57$), albeit the direction of the effect is similar.

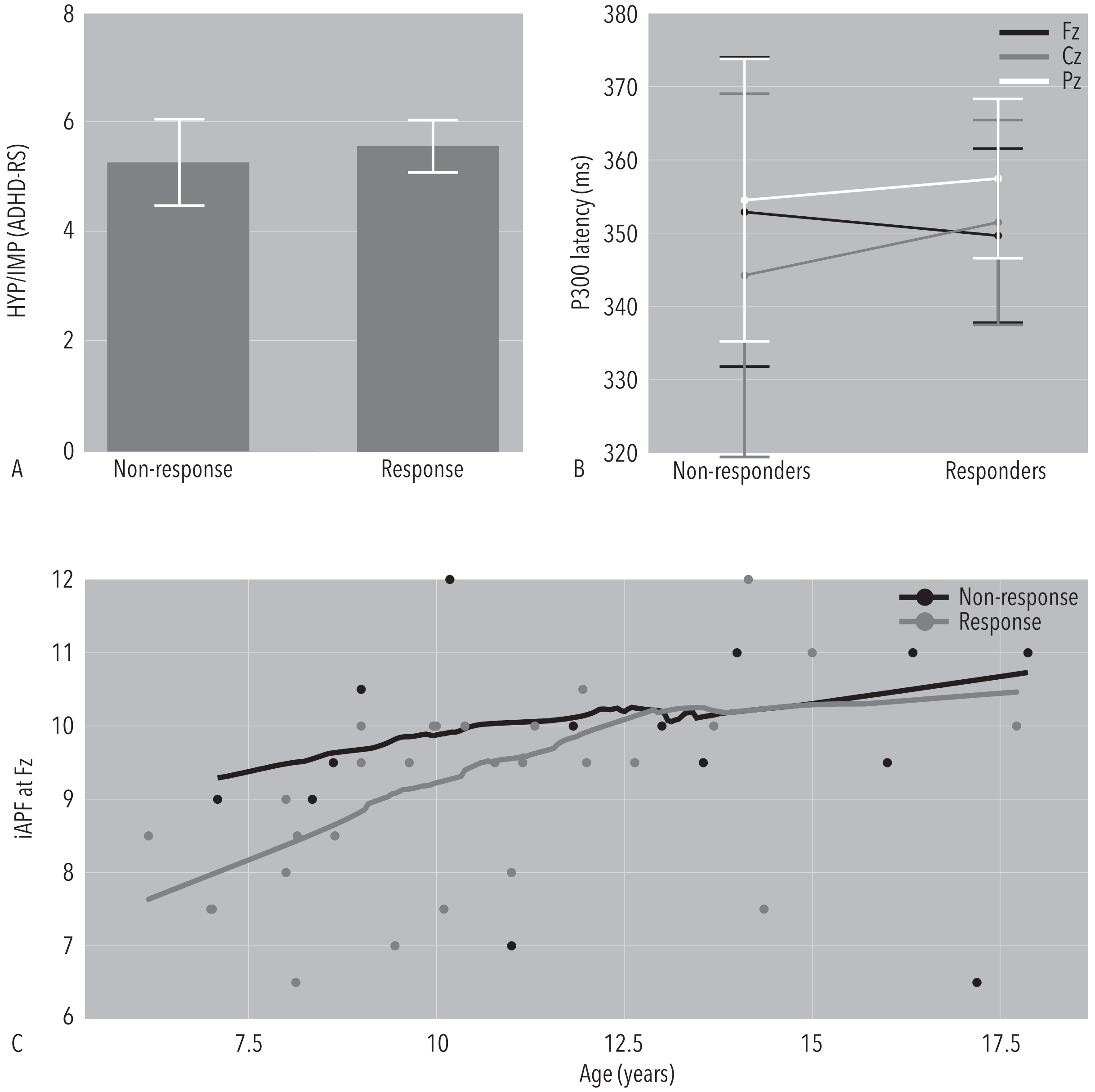

Figure 1A: bar graph of HYP scores at baseline, separated by remission. A GLM Univariate showed that there was no significant difference between responders and non-responders ($F(1,130)=.405$, $p=.526$; $d=-.10$). **1B:** P300 amplitudes (Fz, Cz, Pz) for females only, separated by response. A repeated measures ANOVA showed a non-significant main effect of P300 latency ($F(1,30)=.039$, $p=.845$; $d_{Fz}=.16$, $d_{Cz}=-.21$, $d_{Pz}=-.14$). **1C:** a Loess-fit for iAPF and Age, separated for response and non-response, for boys only. A repeated measures ANOVA showed a non-significant main effect of Response ($F(1,40)=2.512$, $p=.121$; $d_{Fz}=.44$, $d_{FCz}=.29$, $d_{Pz}=.63$, $d_{Oz}=.57$).

Next, sensitivity analyses were performed in SMR group only. A GLM univariate using Remission (remission and no remission) as between-subject factors, age as a covariate, and HYP as a dependent variable yielded a significant effect of Remission (Figure 2A; $F(1,76)=5.237$, $p=.025$; $d=.64$). In the case of P300 analyses, a repeated measures ANOVA using Remission (remission and no remission) as a between-subject factor and Site (Fz, Cz, and Pz) as a within-subject factor was performed in females only. A non-significant main effect of Remission was obtained (Figure 2B; $F(1,19)=1.659$, $p=.213$; $d_{Fz}=.64$, $d_{Cz}=.61$, $d_{Pz}=.16$), although the direction of the effect remained the same. Corroborating the result of a lower iAPF

in boys who remit, a repeated measures ANOVA using Remission as a between-subject factor and Site (Fz, FCz, Pz, and Oz) as a within-subject factor showed a non-significant main effect of Remission (Figure 2C; $F(1,20)=2.665$, $p=.118$; $d_{Fz}=.95$, $d_{FCz}=.82$, $d_{Pz}=.41$, $d_{Oz}=.66$), albeit the direction of the effect was identical to the one found in the main manuscript with a large effect size.

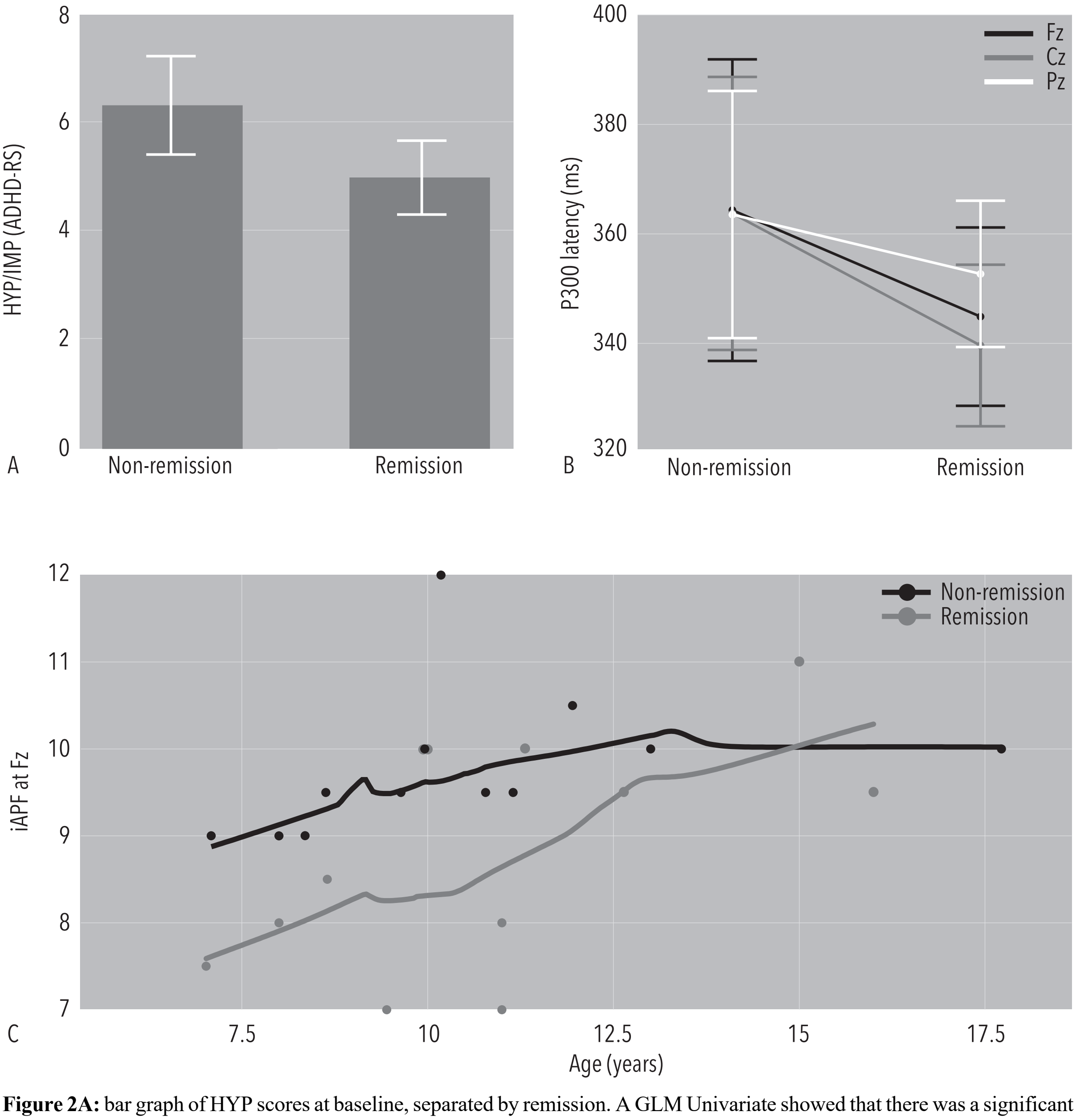

Figure 2A: bar graph of HYP scores at baseline, separated by remission. A GLM Univariate showed that there was a significant difference between remitters and non-remitters ($F(1,76)=.5.237$, $p=.025$; $d=.64$). **2B:** P300 amplitudes (Fz, Cz, Pz) for females only, separated by remission. A repeated measures ANOVA showed a non-significant main effect of P300 latency ($F(1,19)=1.659$, $p=.213$; $d_{Fz}=.64$, $d_{Cz}=.61$, $d_{Pz}=.16$). **2C:** a Loess-fit for iAPF and Age, separated for remission and non-remission, for boys only. A repeated measures ANOVA showed a non-significant main effect of Remission ($F(1,20)=2.665$, $p=.118$; $d_{Fz}=.95$, $d_{Fz}=.82$, $d_{Fz}=.41$, $d_{Fz}=.66$).
